# AURKA inhibition mimics BRCAness

**DOI:** 10.18632/aging.101291

**Published:** 2017-09-11

**Authors:** Jeff Hirst, Andrew K. Godwin

**Affiliations:** Department of Pathology and Laboratory Medicine, University of Kansas Medical Center, Kansas City, KS 66201, USA

**Keywords:** BRCAness, Aurora A kinase, PARP inhibition, synthetic lethality, non-homologous end joining

The role of Aurora Kinase A (AURKA) regulation on mitotic entry, spindle assembly and centrosome function has been well established. Specifically, our past studies have found that increased activity of SRC family kinases promotes tumor invasion and metastasis, and overexpression of the mitotic regulator AURKA drives tumor aneuploidy and chromosomal instability. When combined, Aurora and SRC inhibitors, selectively killed cells that had undergone a preceding aberrant mitosis, and was associated with a postmitotic reattachment defect, and selective removal of aneuploid cell populations [1]. While this study demonstrated the potential of alisertib (MLN8237) as a potential com-bination agent with drugs that regulate DNA structure, the specific activity of AURKA in regulating DNA repair function was previously undefined.

Although it is known that AURKA is overexpressed in ovarian cancer patients and alisertib has potent antigrowth effects in ovarian cancer cell models [2], alisertib showed modest results in clinical studies when used as single agent or in combination with paclitaxel [3]. The limited clinical success of alisertib in ovarian trials led us to pursue a better understanding of AURKA function in regards to DNA stability in order to identify potential therapeutic partners.

In our recent report we find that inhibition of AURKA activity, using either gene silencing or alisertib exposure, increases the error-prone non-homologous end joining (NHEJ) repair pathway [4]. Alisertib treatment promotes increased NHEJ activity and specifically activates DNA-PKcs while decreasing PARP both *in vitro* and *in vivo*. Interestingly, alisertib treatment also impairs the homologous repair pathway (HR) as seen by decreased expression of BRCA1 and BRCA2, increased expression of pH2AX^S139^ levels, and induction of DNA double-strand breaks (DSB).

While the inverse relationship of AURKA and BRCA2 has been previously studied [5], our study shows that AURKA inhibition can mimic “BRCAness” as described above. While the mechanism between AURKA regulation of BRCA2 remains unknown, it suggests an important association that can be exploited for combinatorial therapy. We hypothesized that dual exposure to alisertib and PARP inhibitors would have synergistic effects in both *BRCA*^mut^ and *BRCA*^wt^ cells. While the PARP inhibitor rucaparib (Rubraca) was significantly less active in BRCA competent ovarian cancer cells, it showed a synthetically lethal response in BRCA deficient cells. To support our hypothesis, the combination of alisertib and rucaparib was synergistic in both *BRCA*^wt^ (SKOVIP2) and *BRCA* defective (PEO1) cells. Likewise, NHEJ function was enhanced when using both alisertib and rucaparib, indicating more detrimental DNA damage to the cancer cells. To our knowledge, this was the first study to show synergistic effects of both an AURKA and PARP inhibitor for the treatment of ovarian cancer cells.

**Figure 1:**
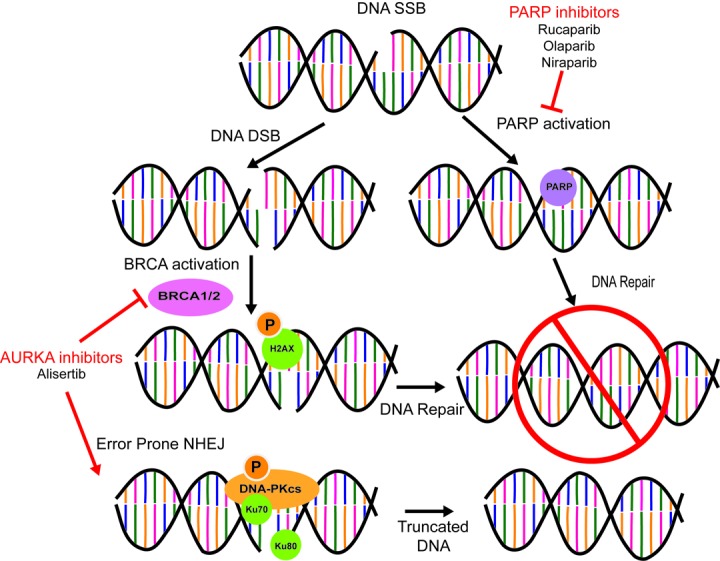
AURKA Inhibition Induces BRCAness and Promotes NHEJ DNA Repair PARP inhibitors (rucaparib, olaparib, niraparib) block DNA SSB repair. AURAK inhibitors (alisertib) blocks BRCA function and promotes error prone NHEJ DNA repair of DNA double strand breaks.

The clinical impact of our study is to broaden the effect of PARP inhibitors for the treatment of ovarian cancer patients. While they have been designated a break-through therapy in breast and ovarian cancer, their application has mostly been limited to use in patients with *BRCA* mutations or other HR pathway defects. In the ovarian cancer population, this limits over half of the patients from receiving this promising breakthrough therapy [6]. The Do et al., study supports the development of combinational therapy between alisertib and rucaparib for potential treatment across both *BRCA* mutant and *BRCA* competent patients. Successful development could open up breakthrough PARP inhibitor therapy for more ovarian cancer patients. Another PARP inhibitor, niraparib (Zejula), was approved in early 2017 for the maintenance treatment of adult patients with recurrent epithelial ovarian, fallopian tube, or primary peritoneal cancer, regardless of *BRCA* mutation status. However, in the Phase III trial there was a vastly superior effect in progression free survival (PFS) for germline BRCA mutant patients when compared to standard of care (22 months vs 9 months) than in BRCA competent patients compared to standard of care (9.3 months vs 3.9 months) [7]. While we specifically studied the combination of rucaparib and alisertib, our data support the use of additional approved PARP inhibitors, *e.g.*, olaparib (Lynparza) or niraparib in combination with alisertib. By mimicking BRCAness, alisertib has the potential to synergize with PARPi's (Figure 1) and potentially improve its clinical efficacy in both BRCA deficient and BRCA competent patients.

Elucidation of AURKA regulating NHEJ and promoting DBS provides novel insight into the function of AURKA and its clinical implication. The development for combinations of AURKA inhibitors, such as alisertib, with PARP inhibitors provides a BRCAness mimic that can target ovarian cancer cells through increased error prone DNA repair pathways.
